# Upper airways colonisation of *Streptococcus pneumoniae* in adults aged 60 years and older: A systematic review of prevalence and individual participant data meta-analysis of risk factors

**DOI:** 10.1016/j.jinf.2020.06.028

**Published:** 2020-10

**Authors:** Emma L. Smith, India Wheeler, Hugh Adler, Daniela M. Ferreira, Raquel Sá-Leão, Osman Abdullahi, Ifedayo Adetifa, Sylvia Becker-Dreps, Susanna Esposito, Helmia Farida, Rama Kandasamy, Grant A. Mackenzie, J. Pekka Nuorti, Susan Nzenze, Shabir A. Madhi, Omar Ortega, Anna Roca, Dodi Safari, Frieder Schaumburg, Effua Usuf, Elisabeth A.M. Sanders, Lindsay R. Grant, Laura L. Hammitt, Katherine L. O'Brien, Prabhu Gounder, Dana J.T. Bruden, Michelle C. Stanton, Jamie Rylance

**Affiliations:** a Department of Clinical Sciences, Liverpool School of Tropical Medicine, Liverpool, United Kingdom; b Instituto de Tecnologia Química e Biológica António Xavier, Universidade Nova de Lisboa, Oeiras, Portugal; c Department of Public Health, School of Health and Human Sciences, Pwani University, Kilifi, Kenya; d Epidemiology and Demography Department, KEMRI-Wellcome Trust Research Programme, Kilifi, Kenya; e Department of Infectious Diseases Epidemiology, London School of Hygiene and Tropical Medicine, Keppel Street, WC1E 7HT, London, United Kingdom; f Department of Paediatrics and Child Health, College of Medicine, University of Lagos, Idi-Araba, Lagos, Nigeria; g Departments of Family Medicine and Epidemiology, University of North Carolina at Chapel Hill, Chapel Hill, NC, United States; h Pediatric Clinic, Department of Surgical and Biomedical Sciences, Università degli Studi di Perugia, Perugia, Italy; i Faculty of Medicine, Diponegoro University, Semarang, Indonesia; j Oxford Vaccine Group, Department of Paediatrics, University of Oxford, Oxford OX3 7LE, United Kingdom; k NIHR Oxford Biomedical Research Centre, Oxford OX3 7LE, United Kingdom; l Medical Research Council The Gambia Unit at LSHTM, Banjul, The Gambia; m Faculty of Infectious and Tropical Diseases, The London School of Hygiene & Tropical Medicine, United Kingdom; n Infection and Immunity Theme, Murdoch Children's Research Institute, Melbourne, Australia; o Department of Paediatrics, University of Melbourne, Melbourne, Australia; p Health Sciences Unit, Faculty of Social Sciences, Tampere University, Finland; q Department of Health Security, National Institute for Health and Welfare (THL), Helsinki, Finland; r Medical Research Council: Respiratory and Meningeal Pathogens Research Unit, University of the Witwatersrand, Johannesburg, South Africa; s Department of Science and Technology/National Research Foundation: Vaccine Preventable Diseases, University of the Witwatersrand, Johannesburg, South Africa; t Gastrointestinal Physiology Laboratory, Department of Surgery, Hospital de Mataró, Universitat Autónoma de Barcelona, Mataró, Spain; u Centro de Investigación Biomédica en Red de enfermedades hepáticas y digestivas (CIBERehd), Instituto de Salud Carlos III, Barcelona, Spain; v Eijkman Institute for Molecular Biology, Jl. Diponegoro no. 69 Jakarta, Indonesia; w Institute of Medical Microbiology, University Hospital Muenster, Muenster, Germany; x Department of Pediatric Immunology and Infectious Diseases, Wilhelmina Children's Hospital, University Medical Center Utrecht, the Netherlands; y Department of International Health, Johns Hopkins Bloomberg School of Public Health, Baltimore, MD, United States; z Arctic Investigations Program, Division of Preparedness and Emerging Infections, Center for Disease Control and Prevention, Anchorage, Alaska; aa Lancaster Medical School, Lancaster University, United Kingdom

**Keywords:** Pneumococcal;  Colonisation;  Adults;  Risk factors

## Abstract

**Background:**

Colonisation with *Streptococcus pneumoniae* can lead to invasive pneumococcal disease and pneumonia. Pneumococcal acquisition and prevalence of colonisation are high in children. In older adults, a population susceptible to pneumococcal disease, colonisation prevalence is reported to be lower, but studies are heterogeneous.

**Methods:**

This is a systematic review and meta-analysis of prevalence of, and risk factors for, pneumococcal colonisation in adults ≥ 60 years of age (PROSPERO #42016036891). We identified peer-reviewed studies reporting the prevalence of *S. pneumoniae* colonisation using MEDLINE and EMBASE (until April 2016), excluding studies of acute disease. Participant-level data on risk factors were sought from each study.

**Findings:**

Of 2202 studies screened, 29 were analysable: 18 provided participant-level data (representing 6290 participants). Prevalence of detected pneumococcal colonisation was 0–39% by conventional culture methods and 3–23% by molecular methods. In a multivariate analysis, colonisation was higher in persons from nursing facilities compared with the community (odds ratio (OR) 2•30, 95% CI 1•26–4•21 and OR 7•72, 95% CI 1•15–51•85, respectively), in those who were currently smoking (OR 1•69, 95% CI 1•12–2•53) or those who had regular contact with children (OR 1•93, 95%CI 1•27–2•93). Persons living in urban areas had significantly lower carriage prevalence (OR 0•43, 95%CI 0•27–0•70).

**Interpretation:**

Overall prevalence of pneumococcal colonisation in older adults was higher than expected but varied by risk factors. Future studies should further explore risk factors for colonisation, to highlight targets for focussed intervention such as pneumococcal vaccination of high-risk groups.

**Funding:**

No funding was required.

## Highlights

• Systematic review and meta-analysis of 18 studies and more than 6000 participants.
• Adults over the age of 60 had a pooled prevalence of pneumococcal carriage of 9%.
• Risk factors: contact with children, smoking and residing in a nursing home.

**Research in context:**

**Evidence before this study:** Nasopharyngeal colonisation with Streptococcus pneumoniae is a pre-requisite for pneumonia and invasive pneumococcal disease. Carriage prevalence in children can be as high as 90%, and geographic location is a strong determinant of rates. In older people, carriage spot-prevalence and risk factors are less clear. Defining these has implications for understanding transmission, for example in nursing home and outbreak settings, and to identify modifiable factors or at-risk groups.

**Added value of this study:** This systematic analysis includes extensive participant level data in adults over 60 years of age to define prevalence and risk factors of upper airway pneumococcal colonisation. We incorporate 18 studies and more than 6000 patients from contrasting geographical and residential settings. Adults over the age of 60 had point prevalence between 0 and 39% using classical microbiology and 3 to 23% using bacterial DNA detection methods. Individuals who have contact with children, smoke and those who reside in a nursing home had a higher prevalence of carriage.

**Implications of all the available evidence:** This evidence is a distillation of previously fragmented data which increase our understanding of population colonisation and risk factors for acquisition. The findings confirm that rates are lower than in children, but identify a significant proportion of individuals which could be protected by developing vaccines which reduce carriage. Using such tools, targeted intervention might reduce the considerable burden of pneumococcal disease in older adults.

Alt-text: Research in context

## Introduction

*Streptococcus pneumoniae* represents a leading cause of morbidity and mortality, resulting from pneumonia, and invasive pneumococcal disease (IPD, encompassing bacteraemia and meningitis). Pneumococcal disease is most common at the extremes of age;^1^, ^2^, ^3^ those over 65 years old are particularly susceptible, in part from the effects of immunosenescence and frequent co-morbidities.^4^ The reported overall incidence of IPD in Europe was 6.2 cases per 100,000 population in 2017.^5^ Higher rates occur in low income countries, although data are fewer*.*^6^, ^7^

*S. pneumoniae* is a frequent transient colonizer of the human upper respiratory tract (naso- and oropharynx). In a minority of cases this can lead to invasive disease but more usually, asymptomatic colonisation generates specific immune responses, and protects against future re-colonisation by the same serotype.^8^, ^9^, ^10^, ^11^, ^12^

Pneumococci spread by droplet transmission of nasopharyngeal bacteria. Young children with high density colonisation are generally considered to be the reservoir for pneumococcal transmission in the population^13^ but adult-to-adult transmission has also been documented, for example in nursing home populations, particularly in outbreak settings.^14^, ^15^ An understanding of population level colonisation and dynamics is important in order to monitor transmission and help to reduce the burden of invasive disease.

Rates of detection of *S.pneumoniae* in the nasopharynx of children are reported to be as high as 90%.^16^, ^17^, ^18^ In ageing adults, rates from highly disparate cohort studies are widely variable. This variation may be driven by study-level factors, including geographical location, site of sampling (nasopharyngeal vs oropharyngeal) and detection methods (e.g., classical microbiological culture in the case of low-density colonisation in adults may be less sensitive than molecular methods). A recent study highlighted a decrease in colonisation density at older ages, particularly >50 years old, and showed improved detection of pneumococcal colonisation when molecular methods were used.^19^ Putative determinants of colonisation include overcrowding, smoking, increasing age, institutionalization, recent respiratory tract infection and contact/cohabitation with young children*.*^1^^,^^20^, ^21^, ^22^, ^23^

The burden of pneumococcal disease has been successfully reduced by the introduction of pneumococcal conjugate vaccination regimens into infant immunisation programmes.^24^ Since the introduction of the seven-valent pneumococcal conjugate vaccine (PCV) in 2000, there has been a general decline in vaccine serotype IPD in children and all other age groups, indicating an indirect effect. This has, however, been accompanied by a concurrent increase in non-vaccine serotype carriage in vaccinated children and adults, resulting in relatively unchanged overall carriage prevalence*.*^11^^,^^17^^,^^25^, ^26^, ^27^, ^28^, ^29^, ^30^

An understanding of population level colonisation and the influence of risk factors is important to assess the risk of acquisition and colonisation and subsequent disease in both the individual and the community. Furthermore, it allows identification of potentially modifiable risk factors to reduce the burden in populations with the highest incidence of IPD (i.e. older adults).

The objective of the systematic review and meta-analysis was to define the prevalence and risk factors for upper airway pneumococcal colonisation in adults over 60 years of age using participant level data from previously published reports.

## Methods

We searched for articles from MEDLINE and EMBASE databases in April 2016 for English language manuscripts published since 1946 using the following search terms: (“pneumococcus” [ti] OR “Streptococcus pneumoniae” [MESH]) AND (“carriage” [ti] OR “colonis* [ti]). Additional articles were identified through discussion with experts with knowledge of other sources. Inclusion and exclusion criteria are given in Box 1.

Two independent reviewers screened the resulting titles and abstracts, reaching agreement through consensus to include studies reporting rates of nasopharyngeal and/or oropharyngeal colonisation of *S. pneumoniae* by detection through classical culture and/or PCR. We searched any adult study, excluding studies which: had no participants over the age of 60 years as documented in the manuscript or by subsequent confirmation with the corresponding author; measured colonisation in participants with acute lower respiratory tract infection or hospitalized participants; lacked peer review or abstract only available; used inappropriate bacterial sampling techniques; lacked clear methodology on population or sampling method; abstract unavailable as English translation. Duplicate articles were removed. Full text articles of the relevant titles/abstracts were evaluated independently by ELS and IW. The review was registered with PROSPERO (#42016036891).

### Assessment of study quality

Study quality data were extracted using the STrengthening the Reporting of Observational studies (STROBE) criteria for epidemiological studies (see Supplementary Table 1).

### Summary data

Data were extracted from original manuscripts into a spreadsheet, disaggregated by age. Where sufficient contextual information was available, we inferred at the study-level pre-defined variables, which were hypothesised or previously documented risk factors for colonisation.

For each included article, we invited the corresponding author to contribute de-identified individual participant level data including potential risk factors for colonisation, and metadata relating to the study. These risk factors were pre-defined by assessing previous research in pneumococcal disease and other respiratory illness. All participants within their respective studies gave their informed written consent to participate in the original study, and each study was approved by their respective ethics committee.

### Data extraction

We extracted information on study country and setting, dates that the study was conducted as well as information regarding pneumococcal vaccination programmes ongoing when the study was conducted. We recorded the number of participants over the age of 60 years as well as the recorded prevalence of *S. pneumoniae* colonisation in that age group, where possible. Where available, we extracted data of the prevalence of carriage across pre-defined risk factors into a spreadsheet'. De-identified participant level data (PLD) were cleaned and imported to R v3.4.3 for analysis.

### Data analysis

We calculated exact binomial confidence intervals around the estimate of proportion colonised for each study. For meta-analysis of all available studies, we generated a generalized linear mixed-effects model of logit transformed proportion using study as a random effect.

For meta-analysis of participant level data, a joint modelling approach with multiple imputation (R package jomo)^31^ was fitted to the colonisation data, to account for ‘missing’ data as a result of differences in the risk factors being collected by each individual study. Models fitted to the data assumed an initial fixed common variance matrix across all individual studies. We used the Markov Chain Monte Carlo (MCMC) algorithm (jomo1ran function) with a burn in of 500,000 and a thinning factor of 1000. Two-hundred imputed datasets were simulated, and a generalised linear mixed model was fitted to each of the simulated datasets, including all imputed risk factors. Imputed estimates of the log-odds associated with each risk factor were combined according to Rubin's rules.^32^

## Results

**Fig. 1:**
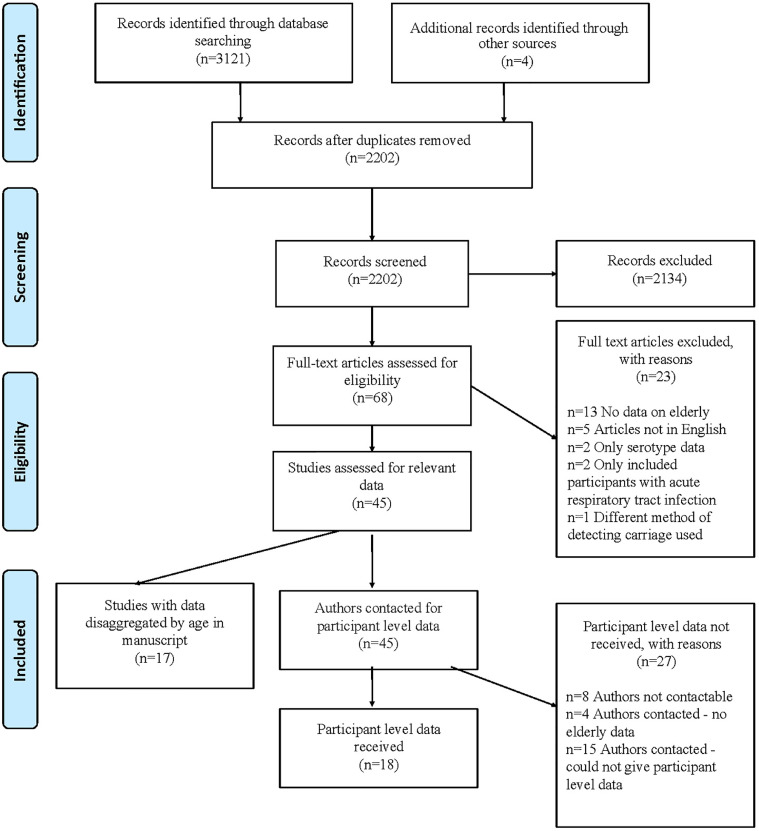
PRISMA flowchart describing the identification and inclusion of data sources.

We screened 2202 studies with 2134 records excluded at this stage (as detailed in Fig. 1). Sixty-eight full text articles were assessed for eligibility of which, 23 studies were excluded due to: (1) no data on elderly participants (*n* = 13); (2) study article unavailable in English (*n* = 5); study contained only data on IPD serotype, not on colonisation (*n* = 2); study included participants with acute respiratory illness (*n* = 2) and; alternative method of detecting carriage used (*n* = 1).

Data were sought from 45 studies, and successfully extracted from 29. Seventeen studies had data disaggregated by age in the manuscript and had relevant data on carriage in participants over 60 years old. We received PLD from 18 studies. In the remaining 27 studies PLD was not obtained due to: authors not contactable (*n* = 8), authors contacted but no data on adults >60 yrs (*n* = 4), authors contacted but could not give PLD for various reasons (*n* = 15). Three further studies could not give PLD but were able to give age-specific prevalence that had not been detailed in the original manuscript.

### Summary-level data

Studies represented varied geographies: Europe (*n* = 13), Africa (*n* = 7), the Americas (*n* = 6), South-East Asia (*n* = 2) and the Western Pacific (*n* = 1). Studies were predominantly from high income countries (*n* = 21), with low income countries presented by two cohorts. Study quality analysis is given in Supplementary Table 1.

**Fig. 2:**
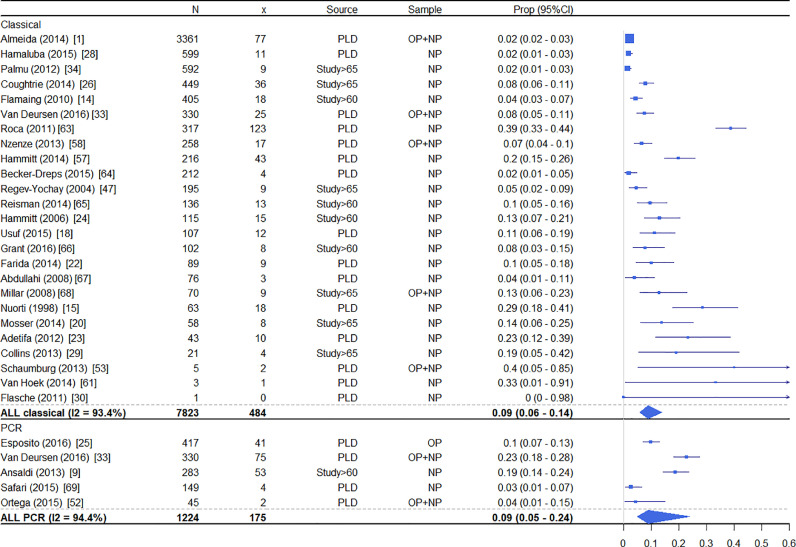
Forest plot of pneumococcal colonisation rates by publication, grouped by country income category. ‘n’ indicates total participants contributing data. ‘data’ indicates participant level data (PLD), or agglomerated result from manuscript which includes adults of ages 60+ or 65+ (Study>60 and Study>65, respectively. ‘site’ indicates nasopharyngeal or oropharyngeal sampling (NP and OP respectively). Proportions colonised are given with exact binomial 95% confidence intervals for each study. Summary using a random effects logistic model with study as the random effect 63, 64, 65, 66, 67, 68, 69.

Prevalence of pneumococcal carriage in included studies was highly variable, ranging from 0%−38•8% in those studies using standard classical culture techniques of naso- and/or oropharyngeal swabs to detect *S. pneumoniae* carriage (Fig. 2). For classical microbiological detection, the pooled estimate of colonisation was 9% (95% confidence interval (CI) 6–14%) representing data from 7823 participants. Using molecular diagnostics like PCR for detection, colonisation prevalence was 2•7%−22•7% (Fig. 2), with pooled estimate of 9% (95% CI 5–24%), representing 1224 individuals. One study compared classical microbiology and PCR detection methods in the same participant group and found significantly higher prevalence when using PCR (7•6%, 95% CI 5•2–10•9 vs 22•7%, 95% CI 18•2–27•2).^33^

**Fig. 3:**
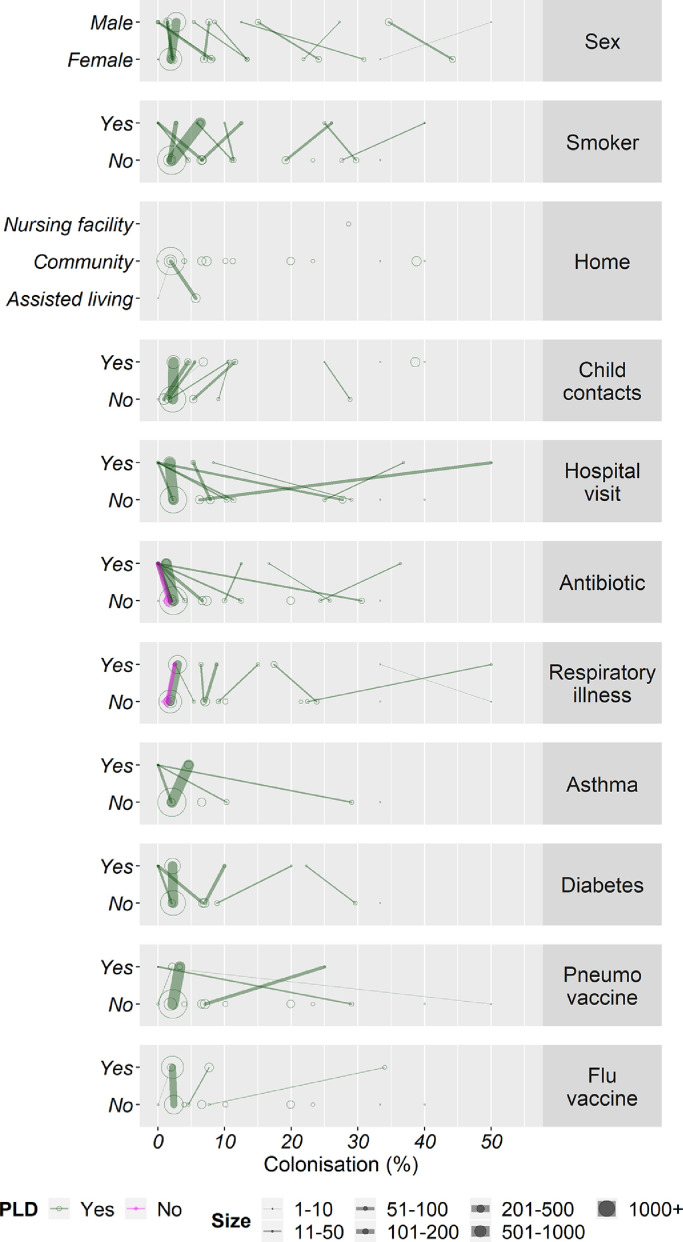
Each panel describes the colonisation rates for a given variable. Studies are indicated by dots which size describes the number of participants contributing data (see key). Lines connect data from the same study where these are available (where dots exist with no line, all participants who were colonised from that study fell into the given category). Graphs indicate studies with participant level data (green circles) and those with only summary data (purple triangles) PLD: Participant Level Data. (For interpretation of the references to colour in this figure legend, the reader is referred to the web version of this article.)

Known and putative risk factors for pneumococcal carriage at the study level are shown in Fig. 3, and in the Supplementary Table 4.

### Participant level data

Participant level data from 6290 participants were available within 18 studies. Studies that used microbiological culture as the method of detection of *S. pneumoniae* carriage were included in a multivariate analysis (5680 participants). Due to many missing values (more than 70% of data), the variables “passive smoking”, “lives with children”, “number sleeping in household”, and “alcohol use” were excluded. The “season” variable was excluded due to inconsistencies in recording the timing of sampling.

In the univariate model, the variables “accommodation”, “setting” and “contact with children” were found to be significantly associated with colonisation (Supplementary Table 4). Those residing in a skilled nursing facility compared to living in the community, those living in a rural area compared to an urban area and those who had regular contact with young children (aged<6 years) were found to have increased rates of pneumococcal colonisation.

The results from the generalised linear mixed model applied to our dataset with imputed values are outlined in Table 1. For all variables used in this analysis, data were obtained in at least 65% of participants. This model showed a significant increase in the prevalence of pneumococcal colonisation in those living in assisted living and more so those in a nursing facility, compared with those living in community (OR 2•30, 95% CI 1•26–4•21 and OR 7•72, 95% CI 1•15–51•85, respectively). Participants from urban areas had significantly lower prevalence of pneumococcal colonisation than did those living in rural settings (OR 0•43, 95%CI 0•27–0•70). Current active smoking and regular (at least weekly) contact with children aged ≤6 years was also associated with significantly higher prevalence of pneumococcal colonisation (OR 1•69, 95% CI 1•12–2•53 and OR 1•93, 95%CI 1•2–2•93 respectively).

**Table 1:** Multiple imputation generalised linear mixed model of risk factors for pneumococcal carriage using participant level data.

Risk factor		Missing data (%)	Odds ratio	95% confidence interval
Age (years)		0	0•99	0•98–1•01
Sex	Female	0•05		
	Male		0•76	0•59–0•98
Accommodation type	Community	0		
	Assisted living		2•30	1•26–4•21
	Nursing facility		7•72	1•15–51•85
Geographical location	Rural	0•07		
	Sub-urban		0•42	0•17–1•02
	Urban		0•43	0•27–0•70
Obstructive lung disease	No	27•3		
	Yes		1•65	0•97–2•81
Asthma	No	32•4		
	Yes		0•71	0•27–1•86
Pneumococcal vaccination*	No	0•09		
	Yes		1•25	0•49–3•23
Smoker	No	2•9		
	Yes		1•69	1•12–2•53
Regular contact with children†	No	10•1		
	Yes		1•93	1•27–2•93
Respiratory illness‡	No	9•1		
	Yes		1•28	0•93–1•74
Received antibiotics in the past 3 months	No	4•1		
	Yes		0•71	0•38–1•32
Climate	Temperate	0		
	Sub-tropical		1•40	0•39–5•07
	Tropical		2•90	0•86–9•76

⁎ Participant has ever received pneumococcal vaccination (pneumococcal polysaccharide vaccine or pneumococcal conjugate vaccine).

† Regular contact was defined by at least weekly contact with a child/children under the age of 6 years.

‡ Any viral or bacterial respiratory illness within the past 2 weeks.

Male participants had significantly lower pneumococcal colonisation than females (OR 0•76, 95% CI 0•59–0•98). Participants’ age was not significantly associated with colonisation.

## Discussion

Our systematic review and meta-analysis incorporate 29 studies and more than 7000 participants from a variety of geographical and residential settings. Adults aged 60 years and older had point prevalence rates of *S. pneumoniae* colonisation between 0% and 38.8% using classical microbiology, and 2.7% and 22•7% using bacterial DNA detection methods. Pooled estimates for both detection techniques were similar (9%), with the three largest studies reporting amongst the lowest rates, notably from European populations.^1^^,^^28^^,^^34^ While substantially lower than rates in paediatric populations, these findings challenge the convention that adults aged over 60 years are only infrequent carriers.

Previous studies have found the average duration of colonisation in *young adults* is 2–3 weeks. ^35^^,^^36^ If colonisation in older populations is similar, the 9% point colonisation rate may have implications for the reservoir of circulating pneumococcus at the community level.  For example, recent vaccine impact studies in the UK have identified a high proportion of invasive disease caused by a few key vaccine-type (e.g. 3, 19A) and non-vaccine type (8, 10A, 15A) pneumococci.^37^ Some of these serotypes (3, 8, 19A) were infrequent/absent in childhood colonisation surveys carried out during the same period,^38^ but have been identified in adult colonisation,^39^ which might explain their persistent circulation.

The most important risk factors were smoking, contact with children and residence in a nursing home compared to living in the community. Living in an urban setting and being male was associated with lower prevalence of carriage. Our findings are consistent with previous studies. Cigarette smoking is a well-documented risk factor for colonisation both directly in the elderly population^1^ and also exposure to passive smoke in children.^40^ Cigarette smoking has also been shown to be a strong independent risk factor for invasive pneumococcal disease in the immunocompetent non-elderly adult population.^41^ Potential mechanistic explanations include the effect of cigarette smoke on the oral microbiome^42^, ^43^, ^44^ with a higher proportion of disease causing organisms and fewer bacteria with interfering capabilities in smokers compared with non-smokers.^45^ Smoking also increases host susceptibility to respiratory viruses through the potential mechanisms of: impaired mucocilary flow and increased permeability of the respiratory epithelium.^46^

Contact with young children, as an independent risk factor for adult colonisation, has been previously documented, and confirms children being the most important reservoir for circulating strains.^41^^,^^47^^,^^48^ Adult-to-adult pneumococcal transmission has also been documented within nursing homes and other institutions, evidenced by cluster analysis of disease outbreaks within these settings.^12^^,^^15^^,^^49^^,^^50^ Our meta-analysis identified nursing home residence as a risk factor, although total participant numbers were small in this subgroup. Findings suggest that this, in combination with reports of nursing home pneumococcal outbreaks, could support the designating of residents as higher-risk and therefore worthy of PCV and pneumococcal polysaccharide vaccine (PPV) vaccination to protect against severe pneumococcal disease. A recent study confirmed that PCV causes a transient reduction in vaccine serotype carriage in older adults^51^, and protection against vaccine serotype pneumonia, further supporting a potential benefit of vaccination of higher-risk groups.

The data demonstrate stark intra-cohort differences in prevalence across different geographic settings, ranging from no documented carriage in a Spanish community setting^52^ to 40% carriage in a community survey in Gabon.^53^ Living in a tropical climate was not identified as an individual risk factor, however the observed higher carriage rates in the tropics may reflect higher population or living densities.^54^^,^^55^ Nonetheless these individual factors may relate only to associations with other aspects of lower economic status. Indeed, in keeping with paediatric data, the increased risk of colonisation associated with residing in a rural setting is partly explained by the economic conditions in these settings, as seen in Aboriginal communities in Australia^21^^,^^56^ and in Sub-Saharan Africa.^57^^,^^58^

Our results derive from rigorous systematic and approved methodology, with strenuous effort to obtain participant-level data. However, the results should be interpreted in light of several considerations. As described, our data was derived from vast geographical settings and as such it is difficult to draw comparisons between such starkly contrasting populations. We used both participant-level data (from 18 studies), and meta-data derived from the study setting and inclusion/exclusion criteria. Not all studies followed the WHO recommendation for measuring nasopharyngeal colonisation.^59^ Our analysis therefore combines nasopharyngeal and oropharyngeal colonisation into a single categorical variable, in order to maximise statistical power. The combination of oro- and nasopharyngeal sampling may have influenced results, incurring a bias towards lower values compared to using nasopharyngeal sampling alone.

Risk factors are necessarily restricted to those for which data were collected (or inferable); some, including alcohol consumption, number of household members and participants’ cohabitation with children had to be excluded due to insufficient completion rates. We were also unable to codify data on seasonality. In order to analyse the remaining variables, we used imputation. Imputation may introduce bias, and although this overall dataset does represent a very large proportion of published data, publication or reporting bias may contribute significantly. There may also be other potential sources of bias in the data as a result of recruitment or sampling methods, which might favour those with certain risk factors for carriage giving a falsely elevated or decreased carriage risk

We have been unable to fully ascertain the association of colonisation with some important risk factors, particularly immunisation. Data on individual elderly participant's pneumococcal vaccination history (with PPV) was included in the meta-analysis and was found to not be a significant factor in pneumococcal colonisation. National paediatric pneumococcal conjugate vaccine policy was not codified into a study variable. This was due to studies being conducted over a number of years, during vaccination policy change, as well as a number of studies in which vaccination policy was not outlined within the journal article. Results from adult populations in high income countries demonstrate reduced vaccine-serotype carriage where paediatric immunisation has been programmatically introduced.^29^^,^^30^^,^^60^^,^^61^ However, in some cohorts from low income countries, the effect on even paediatric circulating serotypes has been suboptimal, demanding better vaccination coverage and time to eradicate PCV serotype circulation.^62^ It may therefore be useful for a longitudinal study to be conducted investigating colonisation in the elderly in the context of childhood vaccination programmes.

We also could not capture potentially significant differences in living environment (for example crowding and paediatric prevalence rates), due to lack of granularity. Data for colonisation densities were unavailable. We recommend that a consistent approach to data capture and reporting is taken in studies; we have suggested a core set of variables which should be considered in the design of such future research (see Box 1). Standardising sampling methods as described by the World Health Organisation^59^, or clarifying the site and diagnostic method within the study protocol is also a priority to enable accurate comparisons to be made between population groups.

**Box 1:** Data elements to include in studies of colonisation rates for *Streptococcus pneumoniae* in older adults*.

**Individual** • Age • Sex • Smoking and passive smoking • Accommodation type (community, institutional facility) • Accommodation density (# per house and per bedroom) • Contact with children (<5 years) in the home • Contact with children (<5 years) at work • Exposure to household air pollution (by specific domestic fuel types) • Antibiotic use (in the preceding 1 month) • Hospital admission (in the preceding 3 m) • Influenza vaccine in the preceding year • Pneumococcal vaccine (type and date) • Vaccination programme in children, time since introduction and coverage • Chronic respiratory illness (COPD, asthma) • Diabetes
**Study** • Sample timing (relative to climate and ‘flu season) • Urban or rural setting • Country pneumococcal vaccination policy

⁎ Based on the appearance in multiple existing studies, or plausible or measurable effect on colonisation to allow future meta-analysis.

Paradoxically, older adults have high incidence rates and case-fatality of pneumococcal disease, despite low observed carriage rates. Given a limited and transient reduction in colonisation after PCV13^51^ in older adults, we need to understand this relationship and the factors which drive progression from colonisation to severe illness. When performing epidemiological and laboratory-based research, this review identifies the core measures which should be consistently reported.

## Declaration of Competing Interest

SBD reports research grants from Pfizer during the conduct of the study and personal fees from Pfizer outside of the submitted work.

PG reports an investigator-initiated grant by Wyeth Pharmaceuticals (now Pfizer) on a pneumococcal colonisation study. The funding agency provided funding support only - they did not provide any input into the study design.

SE reports grants and personal fees from GSK, personal fees from Merck, grants from Abbott, grants and personal fees from Sanofi Aventis, grants and personal fees from Vifor, grants from DMG, outside the submitted work.

GM reports grants from Bill & Melinda Gates Foundation, grants from GAVI the Vaccine Alliance, grants from Pfizer Ltd, during the conduct of a pneumococcal colonisation study.

EAMS reports grants from Wyeth, grants from Pfizer, during the conduct a pneumococcal study

LLH reports grants from Pfizer, GSK, and Merck, outside the submitted work.

LRG reports grants from Pfizer, GSK, and Merck, outside the submitted work and honorarium from Pfizer and Merck.

## Appendix Supplementary materials

Image, application 1

Image, application 2

Image, application 3

Image, application 4
