# Supplementary material for: Upper airways colonisation of Streptococcus pneumoniae in adults aged 60 years and older: A systematic review of prevalence and individual participant data meta-analysis of risk factors
Source: J Infect. 2020 Oct;81(4):540–8. doi: 10.1016/j.jinf.2020.06.028 (PMC7532703; doi:10.1016/j.jinf.2020.06.028)
Supplement: Supplementary file 2 [file mmc2.docx]

**Supplementary Table 2: Details of contact with authors of all eligible journal articles**

| **Paper reference** | **Corresponding author** | **Outcome of contact with authors** |
| --- | --- | --- |
| Abdullahi (2008) | Osman Abdullahi | PLD* received |
| Adetifa (2012) | Ifedayo M. O. Adetifa | PLD received |
| Almeida (2014) | Raquel Sa´-Leao˜ | PLD received |
| Ansaldi (2013) | Paolo Durando | Aggregate data used . Multiple co-authors unable to be contacted for PLD |
| Becker-Dreps (2015) | Sylvia Becker-Dreps | PLD received |
| Collins (2013) | Deirdre A Collins | Authors contacted: not able to share PLD. Aggregate data used |
| Coughtrie (2014) | S C Clarke | Authors contacted: not able to share PLD. Aggregate data used |
| Esposito(2016) | Susanna Esposito | PLD received |
| Farida (2014) | Helmia Farida | PLD received |
| Flamaing (2010) | Not specified | Not able to contact authors. Aggregate data used |
| Flasche (2011) | Stefan Flasche | PLD received |
| Hamaluba (2015) | Rama Kandasamy | PLD received |
| Hammitt (2006) | Laura Hammitt | Authors contacted: not able to share PLD. Aggregate data on elderly participants not available from journal article. Authors able to share aggregate data |
| Hammitt (2014) | Laura Hammitt | PLD received |
| Hansman (1985) |  | Not able to contact authors. No aggregate data in journal article for elderly participants |
| Hill (2006) | Philip C Hill | Authors contacted: not able to share PLD. No aggregate data available on elderly participants in journal article |
| Hussain (2005) | Richard Pebody | Authors contacted: no data for elderly participants. No aggregate data in journal article for elderly participants |
| Krone (2015) | Krzysztof Trzciński | Authors contacted: not able to share PLD. No aggregate data in journal article for elderly participants |
| Lieberman (2005) | David Lieberman | Not able to contact authors. No aggregate data in journal article for elderly participants |
| Mackenzie (2007) | Grant A Mackenzie | Authors contacted: not able to share PLD. No aggregate data in journal article for elderly participants |
| Mackenzie (2010) | Peter Morris | Authors contacted: not able to share PLD. No aggregate data in journal article for elderly participants |
| Memish (2014) | Z.A. Memish | Authors contacted: not able to share PLD. No aggregate data in journal article for elderly participants |
| Millar (2008) | Eugene V. Millar | Authors contacted: not able to share PLD. Aggregate data used |
| Millar (2010) | Eugene V. Millar | Authors contacted: not able to share PLD. No aggregate data in journal article for elderly participants |
| Mosser (2014) | Lindsay R. Grant | Authors contacted: not able to share PLD. Aggregate data used |
| Scott (2016) | Katherine L. O'Brien | Authors contacted: not able to share PLD. Aggregate data the same participants as in Millar et al (2008) |
| Nuorti (1998) | Not specified | PLD received |
| Nzenze (2013) | Shabir A. Madhi | PLD received |
| Nzenze (2014) | Shabir A. Madhi | Authors contacted: no data on elderly participants. No aggregate data in journal article for elderly participants |
| Ortega (2015) | Pere Clavé | PLD received |
| Palmu (2012) | Arto Palmu | Authors contacted: not able to share PLD. Aggregate data used |
| Park (2014) | Wonyong Kim | Not able to contact authors. No aggregate data in journal article for elderly participants |
| Regev-Yochay (2004) | Gili Regev-Yochay | Authors contacted: not able to share PLD. No aggregate data in journal article for elderly participants |
| Reisman (2014) | Jonathan Reisman | Authors contacted: not able to share PLD. Aggregate data on elderly participants not available from journal article. Authors able to share aggregate data |
| Safari (2015) | Dodi Safari | PLD received |
| Schaumburg (2012) | Frieder Schaumburg | PLD received |
| Usuf (2015) | Effua Usuf | PLD received |
| van Deursen (2016) | E.A.M. Sanders | PLD received |
| van Hoek (2014) | Albert Jan van Hoek | PLD received |
| Chi (2003) | David. H Chi | Not able to contact authors. No aggregate data in journal article for elderly participants |
| Roca (2011) | Anna Roca | PLD received |
| Boersma (1993) | Not specified | Not able to contact authors. No aggregate data in journal article for elderly participants |
| Grant (2016) | Lindsay Grant | Authors contacted: not able to share PLD. Aggregate data on elderly participants not available from journal article. Authors able to share aggregate data |
| Brugger (2010) | Kathrin Muhlemann | Authors contacted: no data on elderly participants. No aggregate data in journal article for elderly participants |
| Gunnarsson (1997) | Not specified | Authors contacted: no data on elderly participants. No aggregate data in journal article for elderly participants |

*Participant level data
