# Supplementary material for: Upper airways colonisation of Streptococcus pneumoniae in adults aged 60 years and older: A systematic review of prevalence and individual participant data meta-analysis of risk factors
Source: J Infect. 2020 Oct;81(4):540–8. doi: 10.1016/j.jinf.2020.06.028 (PMC7532703; doi:10.1016/j.jinf.2020.06.028)
Supplement: Supplementary file 4 [file mmc4.docx]

**Supplementary table 4: Univariate model of risk factors for pneumococcal carriage using participant level data**

| **Risk factor** | **Missing data (number)** | **Missing data (%)** | **Generalised linear mixed model (univariate) result** |
| --- | --- | --- | --- |
| Age | 0 | 0.00 | Non-significant (linear) |
| Sex | 3 | 0.05 | Non-significant |
| Accommodation type* | 0 | 0.00 | Significant |
| Setting† | 4 | 0.07 | Significant |
| Obstructive lung disease | 1611 | 27.31 | Borderline |
| Asthma | 1918 | 32.38 | Borderline |
| Pneumococcal vaccination‡ | 551 | 0.09 | Non-significant |
| Influenza vaccination | 1146 | 19.37 | Non-significant |
| Smoker | 170 | 2.94 | Borderline |
| Passive smoker | 4530 | 76.69 | Non-significant |
| Lives with children§ | 4385 | 74.30 | Significant |
| Contact with children¶ | 599 | 10.18 | Significant |
| Number sleeping in the same room | 5429 | 91.99 | Non-significant |
| Respiratory illness (within two weeks) | 535 | 9.16 | Borderline |
| Antibiotics (within three months) | 241 | 4.14 | Non-significant |
| Alcohol use | 5552 | 94.00 | Non-significant |
| Climate | 0 | 0.00 | Non-significant |
| Season | 269 |  | Not comparable |
| Number living in the household | 1389 | 23.47 | Borderline |

The cells shaded in orange highlight the variables that were excluded from subsequent analysis due to >50% of data missing. The row highlighted in red highlights the “season” variable. This was excluded from the analysis due to the data recorded being not comparable.

*Accommodation defined as from a “skilled nursing facility” or from the “community”.

†Setting defined as “urban”, “sub-urban” or “rural”.

‡ Participant has ever received pneumococcal vaccination (pneumococcal polysaccharide vaccine or pneumococcal conjugate vaccine)

§Living with children <6 years old.

¶Contact with children defined as at least weekly contact with a child <6 years old.
